# Identification of *UBAP1* mutations in juvenile hereditary spastic paraplegia in the 100,000 Genomes Project

**DOI:** 10.1038/s41431-020-00720-w

**Published:** 2020-09-15

**Authors:** Thomas Bourinaris, Damian Smedley, Valentina Cipriani, Isabella Sheikh, Alkyoni Athanasiou-Fragkouli, Patrick Chinnery, Huw Morris, Raquel Real, Victoria Harrison, Evan Reid, Nicholas Wood, Genomics England Research Consortium, Jana Vandrovcova, Henry Houlden, Arianna Tucci

**Affiliations:** 1 Department of Neuromuscular Disorders, UCL Institute of Neurology, Queen Square, London, UK; 2 William Harvey Research Institute, Barts and The London School of Medicine and Dentistry, Queen Mary University of London, Charterhouse Square, London, EC1M 6BQ UK; 3 Department of Clinical Neurosciences, School of Clinical Medicine, University of Cambridge, Cambridge Biomedical Campus, Cambridge, UK; 4 Medical Research Council Mitochondrial Biology Unit, University of Cambridge, Cambridge Biomedical Campus, Cambridge, UK; 5 Department of Clinical and Movement Neurosciences, UCL Institute of Neurology, Queen Square, London, UK; 6 Wessex Clinical Genetics Service, Princess Anne Hospital, Southampton, SO16 5YA UK; 7 Department of Medical Genetics and Cambridge Institute for Medical Research, University of Cambridge, Cambridge, UK

**Keywords:** Genetics research, Neurological disorders

## Abstract

Hereditary spastic paraplegia (HSP) is a group of heterogeneous inherited degenerative disorders characterized by lower limb spasticity. Fifty percent of HSP patients remain yet genetically undiagnosed. The 100,000 Genomes Project (100KGP) is a large UK-wide initiative to provide genetic diagnosis to previously undiagnosed patients and families with rare conditions. Over 400 HSP families were recruited to the 100KGP. In order to obtain genetic diagnoses, gene-based burden testing was carried out for rare, predicted pathogenic variants using candidate variants from the Exomiser analysis of the genome sequencing data. A significant gene-disease association was identified for *UBAP1* and HSP. Three protein truncating variants were identified in 13 patients from 7 families. All patients presented with juvenile form of pure HSP, with median age at onset 10 years, showing autosomal dominant inheritance or de novo occurrence. Additional clinical features included parkinsonism and learning difficulties, but their association with *UBAP1* needs to be established.

## Introduction

Hereditary spastic paraplegia (HSP) represents a wide spectrum of rare inherited neurodegenerative conditions, typically characterized by progressive limb spasticity. HSP is traditionally classified into pure and complicated forms, depending on the presence of additional clinical features, such as cognitive decline, cerebellar ataxia, peripheral neuropathy, or parkinsonism. To date, more than 80 genes have been associated with HSP under various modes of inheritance, including autosomal recessive, autosomal dominant (AD), or X-linked [1].

Recent advances in molecular analysis techniques have led to the identification of new genetic loci associated with HSP. Yet, for up to 70% of cases clinically diagnosed as HSP the genetic cause remains unknown [2].

The 100,000 Genomes Project (100KGP) was established to deliver advanced genomic testing for NHS patients with rare disease and cancer. One of the aims of the rare disease arm of the project was to make molecular diagnoses and novel gene discoveries through whole genome sequencing. Over 60,000 participants with genetically undiagnosed rare disease have been recruited [3], including over 400 HSP families.

Here, we describe 13 patients from 7 families with HSP recruited to the 100KGP, who carry 3 truncating variants in *UBAP1*, a newly identified gene that causes pure forms of juvenile HSP [4]. Variant interpretation analysis had been initially carried out via the 100KGP automated pipeline in known HSP genes [5], and failed to identify a genetic diagnosis in all families. An ongoing re-analysis of the data in the research environment using gene-based burden testing for rare, predicted damaging variants allowed the rapid identification of the three pathogenic *UBAP1* variants in seven families.

## Materials and methods

### Patients

Following ethical approval (14/EE/1112), consenting participants were recruited to the 100KGP cohort. Standardized baseline clinical data were recorded at the time of recruitment using the Human Phenotyping Ontology, according to the HSP data model (Supplementary information). Further, additional information was collected retrospectively by contacting the recruiting clinician for each patient (summarized in Table 1). Patients had been previously tested negative for single nucleotide variants, small deletions/insertions and CNVs in genes from the PanelApp [5] curated virtual gene panel for HSP. CNV calls were generated by Genomics England using Manta and Canvas software (Illumina) and processed using custom R scripts to detect CNVs overlapping the *SPAST* gene [5]. No likely pathogenic variants were identified.

**Table 1:** Detailed clinical characterization of the individuals included in the study and carrying truncating mutations in *UBAP1* (NM_016525.4; NP_057609.2).

Family/ID	F1-II.1	F2-II.1	F3-II.1	F4-II.1	F5-I.2	F5-II.1	F5-II.2	F5-II.4	F6-I.2	F6-II.1	F6-II.2	F7-II.1	F7-II.2
Variant	c.426_427del; p.(Lys143SerfsTer15)	c.426_427del; p.(Lys143SerfsTer15)	c.535G > T; p.(Glu179Ter)	c.373C > T; p.(Gln125Ter)	c.535G > T; p.(Glu179Ter)	c.535G > T; p.(Glu179Ter)	c.535G > T; p.(Glu179Ter)	c.535G > T; p.(Glu179Ter)	c.535G > T; p.(Glu179Ter)	c.535G > T; p.(Glu179Ter)	c.535G > T; p.(Glu179Ter)	c.535G > T; p.(Glu179Ter)	c.535G > T; p.(Glu179Ter)
CADD score (phred)	29.8	29.8	37	36	37	37	37	37	37	37	37	37	37
Sex	M	M	M	M	F	M	F	F	F	F	M	M	F
Ethnicity	Caucasian	Caucasian	Caucasian	Ashkenazi	Caucasian	Caucasian	Caucasian	Caucasian	Caucasian	Caucasian	Caucasian	Caucasian	Caucasian
Family history	De novo	De novo	Positive	Positive	Positive	Positive	Positive	Positive	Positive	Positive	Positive	Positive	Positive
Age at examination (years)	16	69	33	43	42	23	24	18	n/a	36	n/a	10	11
Age at onset (years)	9	9	18	11	11	10	10	9	n/a	30	11	9	7
Develpoment	Normal. Toe walker	Normal	Normal	Normal	Normal	Normal	Normal	Normal	Normal	Normal	Normal	Normal	Normal
Presenting symptom(s)	Gait difficulty, tiptoe walking	Difficulty running	Mild stumbling	Gait difficulty	Gait difficulty	Gait difficulty	Difficulty running	Gait difficulty, frequent falls	n/a	n/a	n/a	Gait difficulty, falls, foot pain	Unsteady gait, falls, ankle pain
Lower limb power	4+/5 proximal, 4/5 distal	Reduced in a pyramidal distribution	Mild pyramidal weakness	Pyramidal weakness	Reduced	Moderate weakness	Reduced	Normal	n/a	Normal	Normal	Normal	Normal
Lower limb spasticity	Markedly present	Present	Present	Markedly present	Present	Present	Present	Present	n/a	No	Present	Present	Present
Lower limb reflexes	Very brisk, sustained ankle clonus	Brisk, sustained ankle clonus	Increased, sustained ankle clonus	Increased	Increased	Increased, sustained ankle clonus	Increased	Increased	n/a	Very brisk	Very brisk	Increased, mild clonus	Increased, clonus
Plantar reflex	Withdrawal	Extensor	Extensor	Extensor	n/a	Extensor	n/a	Extensor/withdrawal	n/a	Extensor	Extensor	n/a	Extensor
Upper limb power	Normal	Normal	Normal	Normal	Normal	Normal	Normal	Normal	n/a	Normal	Normal	Normal	Normal
Upper limb tone	Normal	Extrapyramidal rigidity	Normal	Normal	Normal	Normal	Normal	Normal	n/a	Normal	Normal	Normal	Normal
Upper limb reflexes	Normal	n/a	Normal	Brisk	Normal	Normal	Normal	Normal	n/a	Normal	Absent	Normal	Normal
Bladder dysfunction	No	Suprapubic catheter	Urinary urgency	No	Urinary urgency	No	No	No	n/a	no	no	no	Urinary urgency
Sensory dysfunction	No	No	No	No	No	No	No	No	n/a	No	distal numbness	No	No
Cognitive impairment	No	No	No	No	No	No	No	No	n/a	No	No	No	No
Ataxia	No	No	No	No	No	No	No	No	n/a	No	No	No	No
Dysarthria	No	No	No	No	No	No	No	No	n/a	No	No	No	No
Additional features	None	Parkinsonism	Occasional cramps	None	Cramps	Learning difficulties, spasms	Learning difficulties	None	n/a	Strabismus	n/a	Severe bilateral valgus foot deformity	None
Progression	Wheelchair use after 2–3 years	Wheelchair dependant from age 55	Walks without aids	Walks without aids	Walks with a single crutch	Walks without aids	Walks without aids	Walks without aids	n/a	Walks without aids	Walks without aids	Walks with crutches, occasionally uses wheelchair	Walks with crutches
Imaging	MRI brain and spine normal	MRI brain: mild generalized cortical subcortical volume loss	MRI brain normal	MRI brain normal	n/a	n/a	n/a	n/a	n/a	MRI brain normal	n/a	n/a	n/a

*M* male, *F* female, n/a not available.

### Gene-based burden testing

A statistical analysis framework was developed to detect enrichment of rare, predicted pathogenic variants in novel genes for specific diseases. The analysis was run on all rare, coding variants that segregated with disease as expected for each possible mode of inheritance (minor allele frequencies, MAF < 0.1% dominant, <1% recessive), identified from Exomiser [6, 7] analysis of each case’s whole genome data. The following case-control association analysis framework was used: “case” sets were defined as all 100KGP probands recruited under HSP, while corresponding “control” sets were all recruited 100KGP probands except those recruited under a neurological disorder. We tested for enrichment of rare variants using Fisher’s exact test under four scenarios: (1) rare, predicted pathogenic variants (Exomiser variant score > 0.8 corresponding to rare variants that are predicted to be pathogenic by in silico prediction tools REVEL [8] and/or MVP [9]), (2) rare variants in a constrained coding region [10], (3) rare, predicted loss of function (LoF) variants, (4) rare, de novo variants. For the latter, only trios or larger families where de novo calling was possible were considered. The Benjamini and Hochberg method was used to correct for multiple testing; an overall false discovery rate adjusted *q* value threshold of 0.10 was used for claiming significant gene-disease associations.

All variants described in this study have been submitted to ClinVar.

## Results

In the context of an ongoing gene-based burden analysis of the rare disease component of the 100KGP data, a significant gene-disease association was identified for *UBAP1* and HSP. A significant excess of LoF variants was observed in 5 out of 417 HSP cases compared to 16,449 non-neurological 100KGP probands used as controls (odds ratio, OR = 66.5; *q* value = 0.002). Through further inspection of newly available 100KGP data and other neurological probands that were excluded from the case-control analysis dataset, we identified two additional patients with spastic paraplegia and UBAP1 truncating variants. One of these patients had been recruited under HSP diagnosis and the other as familial Parkinson’s disease (PD), but was also affected by HSP.

**Fig. 1:**
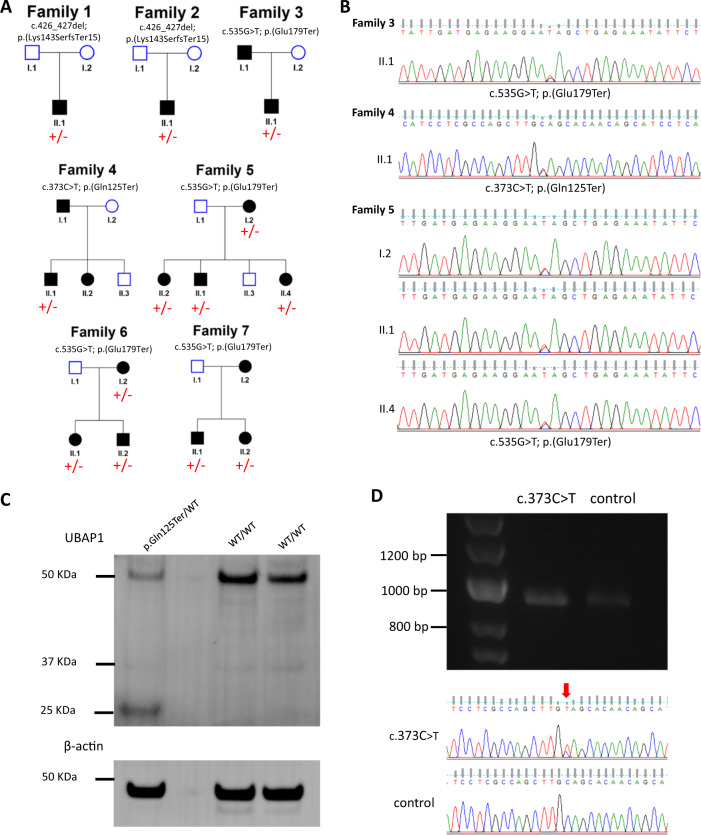
Pedigrees, DNA, RNA and protein analysis of UBAP1. **a** Pedigrees, **b** Sanger Sequencing, **c** Western Blot, **d** RNA. **a** Pedigrees of families reported in this study. Squares represent males, circles represent females, shaded shapes represent individuals with HSP, and unshaded shapes represent individuals without HSP. **b** Electropherograms of Sanger sequencing performed for the validation of variants identified in families 3, 4, and 5. **c** Western Blot analysis for the proband of family 4 (p.Gln125Ter/WT) and two healthy controls (WT/WT), showing reduced expression of normal size ubiquitin-associated protein 1 (UBAP1) and the presence of a smaller additional band in the proband, indicating the presence of truncated protein. Loading control with β-actin.was performed to confirm that the same amount of the total protein was loaded for all three samples. **d** Gel electrophoresis of PCR products amplified from cDNA synthesized from RNA of the proband of family 4 (c.373C > T) and a healthy control, showing the presence of amplicons of target sequence in both samples. Sanger sequencing of the same PCR products confirm the result as well as the presence of the variant in the proband, as shown by the respective electropherograms. WT wild type; KDa kilodalton; bp base pairs.

In total, seven families carrying three different truncating variants in *UBAP1* were identified in the 100KGP data (Table 1, ClinVar SUB7456898). Families 1 and 2 were consistent with de novo occurrence of HSP (Fig. 1a), while the pedigrees of families 3–7 suggested an AD pattern of inheritance (Fig. 1a) that was confirmed by segregation analysis in family 5 (Fig. 1b). The identified variants included: c.426_427del; p.(Lys143SerfsTer15) in two families, c.535G > T; p.(Glu179Ter) in four families and c.373C > T; p.(Gln125Ter) in one family (NM_016525.4; NP_057609.2) (Fig. 1a).

The median age at onset was 10 years (interquartile range 9–11 years). In most cases the onset occurred during childhood, with some individuals having a later age at onset, including early adulthood. The latest age at onset was 30 years (Table 1).

Overall, clinical history and examination of the 13 individuals were consistent with uncomplicated HSP phenotype. The first symptoms included difficulties with running and walking, with frequent falls and leg scissoring also reported. Disease course was similar in all families, with slowly progressive lower limb spasticity and weakness, except proband of family 1, who progressed over 2–3 years to wheelchair use. Additional symptoms included learning difficulties in family 5, reduced light touch in the lower limb distally and upper limb areflexia in one of the affected members of family 6, although no results of nerve conduction studies were available. Intrafamilial heterogeneity was observed, with a milder phenotype usually associated with a later age of onset. At the time of examination, the most frequent signs included lower limb weakness, extensor plantars and brisk reflexes of the lower limbs. Only the proband of family 4 presented brisk upper limb reflexes, while none of the individuals of our cohort had spasticity or weakness of the upper limbs. Bladder dysfunction, mainly presenting with urinary urgency, was also reported in three individuals.

Interestingly, in two of the families an extrapyramidal syndrome consistent with typical Parkinson Disease presented later on in the disease course. The proband of family 2 was diagnosed with levodopa-responsive PD in his mid-1950s, with predominantly left-sided rest tremor, bradykinesia and cogwheel rigidity particularly in the upper limb, facial hypomimia, and normal eye movements. The DaTscan was reported abnormal for bilaterally reduced uptake of the tracer in the striatum. The father of the proband of family 3, who was also affected by HSP, was reported to have PD with onset in early 1970s. Each family carried a different pathogenic variant. The size of this analysis does not allow to correlate the presence of parkinsonism with the identified pathogenic variants in *UBAP1*.

A Western Blot for the proband of family 4, carrying a heterozygous c.373C > T; p.Gln125Ter variant, revealed the presence of truncated protein along with reduced amount of normal size protein (Fig. 1c). PCR of c.DNA, synthesized from RNA isolated from the same individual, showed that mRNA escapes nonsense mediated decay (Fig. 1d), as previously reported [4].

## Discussion

Here, we describe the genetic and clinical features of a cohort of UK HSP patients due to UBAP1 protein truncating variants. The 100KGP is a research project to implement WGS in a national healthcare system. Patient with a clinical diagnosis of HSP were recruited from local hospitals in England. In most cases, the relevant HSP genes were sequenced prior to recruitment. This dataset represents one of the largest HSP cohort with WGS data from a single country. In this dataset the prevalence of UBAP1 in HSP is 1.7% (7 families out of 417).

Ubiquitin-associated protein 1 (encoded by *UBAP1*) constitutes a subunit of mammalian endosomal sorting complex required for transport I (ESCRT-I) and is involved in endosomal dynamics in neurons. Furthermore, it is involved in proteasomal degradation of ubiquitinated proteins. Both cellular pathways have been involved in HSP and our study further confirms the importance of such pathways in this disease [11].

While we were conducting this study, truncating *UBAP1* variants were reported in HSP families of diverse geographic origin, including the three variants we are reporting in this study [4, 12–14]. Thus far, and including our study, 30 families with AD HSP have been reported (Supplementary Table 1). The majority of the patients present with a pure form of juvenile-onset HSP. Of all cases reported, the average age at onset of symptoms was 9 years (interquartile range 7–11 years). Adult onset has been reported in two cases only (onset at age 30 and 61, respectively). Although a pure HSP phenotype has been reported in most families, two of the previously reported families additionally presented with cerebellar symptoms, while two of the families reported in this study presented with symptoms typical of an extrapyramidal disorder, this was later on in the disease course. While most families present with positive family history, de novo occurrence is reported in seven families. Incomplete penetrance has been described in three families.

**Fig. 2:**
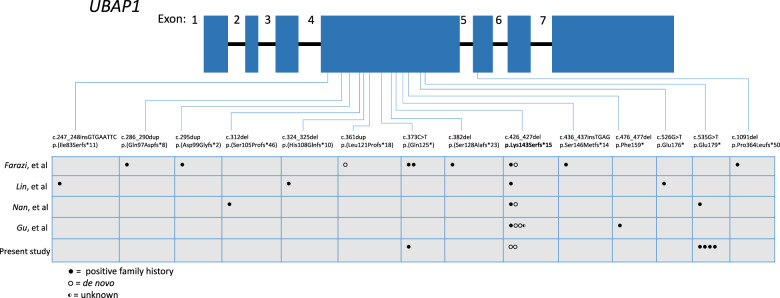
Schematic representation of *UBAP1* with all variants reported in this study and previously. The table indicates the number of families carrying each variant. Each circle represents one family. Shaded circles represent positive family history, unshaded circles represent de novo occurrence, and half-shaded circles indicate unknown family history. Exons are numbered like in Fard et al. 2019 [4].

In total, 14 different truncating variants have been reported so far. All but one of these variants occur within exon 4 of *UBAP1* (Fig. 2). The most frequent mutation identified so far by all studies is the p.(Lys143Serfs*15), reported in 11 families out of 30 across different populations. In the current study, representing HSP in the British population, the most frequent mutation is p.(Glu179Ter), only previously described in one family in the Japanese population [13].

Our study provides further evidence that *UBAP1* causes mostly pure forms of AD HSP. Among the known genes that cause pure forms of HSP, with 30 families described so far, *UBAP1* is a common cause of AD juvenile pure HSP.

## Supplementary information

Suppl

Supplementary Table 1
