# Supplementary material for: Identification of UBAP1 mutations in juvenile hereditary spastic paraplegia in the 100,000 Genomes Project
Source: Eur J Hum Genet. 2020 Sep 15;28(12):1763–8. doi: 10.1038/s41431-020-00720-w (PMC7784862; doi:10.1038/s41431-020-00720-w)
Supplement: Supplementary file 2 — Supplementary Table 1 [file 41431_2020_720_MOESM2_ESM.docx]

**SUPPLEMENTARY INFORMATION**

**The members of The Genomics England Research Consortium are**:

J. C. Ambrose^1^, P. Arumugam^1^, E. L. Baple^1^, M. Bleda^1^, F. Boardman-Pretty^1,2^, J. M. Boissiere^1^, C. R. Boustred^1^, H. Brittain^1^, M. J. Caulfield^1,2^, G. C. Chan^1^, C. E. H. Craig^1^, L. C. Daugherty^1^, A. de Burca^1^, A. Devereau^1^, G. Elgar^1,2^, R. E. Foulger^1^, T. Fowler^1^, P. Furió-Tarí^1^, J. M. Hackett^1^, D. Halai^1^, A. Hamblin^1^, S. Henderson^1,2^, J. E. Holman^1^, T. J. P. Hubbard^1^, K. Ibáñez^1, 2^, R. Jackson^1^, L. J. Jones^1,2^, D. Kasperaviciute^1,2^, M. Kayikci^1^, L. Lahnstein^1^, K. Lawson^1^, S. E. A. Leigh^1^, I. U. S. Leong^1^, F. J. Lopez^1^, F. Maleady-Crowe^1^, J. Mason^1^, E. M. McDonagh^1,2^, L. Moutsianas^1,2^, M. Mueller^1,2^, N. Murugaesu^1^, A. C. Need^1,2^, C. A. Odhams^1^, C. Patch^1,2^, D. Perez-Gil^1^, D. Polychronopoulos^1^, J. Pullinger^1^, T. Rahim^1^, A. Rendon^1^, P. Riesgo-Ferreiro^1^, T. Rogers^1^, M. Ryten^1^, K. Savage^1^, K. Sawant^1^, R. H. Scott^1^, A. Siddiq^1^, A. Sieghart^1^, D. Smedley^1,2^, K. R. Smith^1,2^, A. Sosinsky^1,2^, W. Spooner^1^, H. E. Stevens^1^, A. Stuckey^1^, R. Sultana^1^, E. R. A. Thomas^1,2^, S. R. Thompson^1^, C. Tregidgo^1^, A. Tucci^1,2^, E. Walsh^1^, S. A. Watters^1^, M. J. Welland^1^, E. Williams^1^, K. Witkowska^1,2^, S. M. Wood^1,2^, M. Zarowiecki^1^

1. Genomics England, London, UK
2. William Harvey Research Institute, Queen Mary University of London, London, EC1M 6BQ, UK

**Clinical data collection**

For each patient recruited under hereditary spastic paraplegia, HPO terms were used to collect phenotypic data, according to the table below (<https://www.genomicsengland.co.uk/about-genomics-england/the-100000-genomes-project/information-for-gmc-staff/rare-disease-documents/>).

| Abnormality of higher mental function (HP:0011446) | Cognitive impairment (HP:0100543) | Psychosis (HP:0000709) |
| --- | --- | --- |
| Neurological speech impairment (HP:0002167) | Dysarthria (HP:0001260) | Dysphagia (HP:0002015) |
| Abnormality of the eye (HP:0000478) | Cataract (HP:0000518) | Congenital cataract (HP:0000519) |
| Optic atrophy (HP:0000648) | Abnormality of eye movement (HP:0000496) | Spasticity (HP:0001257) |
| Abnormal pyramidal signs (HP:0007256) | Hyperreflexia in upper limbs (HP:0007350) | Upper limb spasticity (HP:0006986) |
| Skeletal muscle atrophy (HP:0003202) | Fasciculations (HP:0002380) | Proximal upper limb amyotrophy (HP:0008948) |
| Proximal lower limb amyotrophy (HP:0008956) | Distal upper limb amyotrophy (HP:0007149) | Distal lower limb amyotrophy (HP:0008944) |
| Ataxia (HP:0001251) | Gait ataxia (HP:0002066) | Limb ataxia (HP:0002070) |
| Abnormality of the basal ganglia (HP:0002134) | Dystonia (HP:0001332) | Parkinsonism (HP:0001300) |
| Myoclonus (HP:0001336) | Sensory impairment (HP:0003474) | Impaired vibratory sensation (HP:0002495) |
| Impaired tactile sensation (HP:0010830) | Impaired proprioception (HP:0010831) | Impaired temperature sensation (HP:0010829) |
| Impaired pain sensation (HP:0007328) | Abnormality of central motor conduction (HP:0012079) | Motor axonal neuropathy (HP:0007002) |
| Demyelinating motor neuropathy (HP:0007220) | Distal peripheral sensory neuropathy (HP:0007067) | Sensory axonal neuropathy (HP:0003390) |
| Demyelinating sensory neuropathy (HP:0011402) | Mixed demyelinating and axonal polyneuropathy (HP:0007327) | Hypoplasia of the corpus callosum (HP:0002079) |
| Focal white matter lesions (HP:0007042) | Cerebral atrophy (HP:0002059) | Cerebellar atrophy (HP:0001272 |

.
